# Post-Orthodontic Lower Incisors Recessions: Combined Periodontic and Orthodontic Approach

**DOI:** 10.3390/ijerph17218060

**Published:** 2020-11-02

**Authors:** Ilan Beitlitum, Vered Barzilay, Fatma Rayyan, Alon Sebaoun, Rachel Sarig

**Affiliations:** 1 Department of Periodontology and Dental Implantology, The Maurice and Gabriela Goldschleger School of Dental Medicine, Sackler Faculty of Medicine, Tel Aviv University, Tel Aviv 6997801, Israel; beilan1@bezeqint.net (I.B.); alon.sebaoun@gmail.com (A.S.); 2 Department of Orthodontics, The Maurice and Gabriela Goldschleger School of Dental Medicine, Sackler Faculty of Medicine, Tel Aviv University, Tel Aviv 6997801, Israel; vered.barzilay@gmail.com; 3 Department of Oral Biology, The Maurice and Gabriela Goldschleger School of Dental Medicine, Sackler Faculty of Medicine, Tel Aviv University, Tel Aviv 6997801, Israel; fatma_rayyan@hotmail.com; 4 The Dan David Center for Human Evolution and Biohistory Research, Sackler Faculty of Medicine, Tel-Aviv University, Tel Aviv 6997801, Israel

**Keywords:** lingual retainer, gingival recession, connective tissue graft, orthodontics, root coverage

## Abstract

The bonded lingual retainer (BLR) is considered a favorable choice for retaining lower incisors’ alignment post-orthodontic treatment; however, it may cause some unwanted effects such as inadvertent tooth movement and torque changes. These often result in gingival recession (Miller class III-type) with exposure of the root surface, which compromises the esthetics and hinders the comfort of the patient. Fifteen post-orthodontic patients presenting Miller class III-type recessions with BLR were examined. Two protocols were used: the first included the removal of the BLR prior to surgery and the second included only a surgical approach. All patients underwent the same surgery of a modified tunnel double papilla procedure for root coverage. The gingival recession was measured using a dental probe before, and three to six months post-surgery. The average improvement in recession depth was significantly greater (*p* = 0.008) for the protocol that included removal of the BLR (4.0 ± 0.83 mm) with an improvement of 87.2% as compared to the second protocol that showed an improvement of 43.8% (1.88 ± 1.29 mm). Removing the BLR prior to surgery is beneficial for predictable root coverage in post-orthodontic Miller class III recessions.

## 1. Introduction

Long-term stability is one of the main challenges of orthodontic treatment [1,2]. Several protocols were suggested to maintain the alignment of mandibular canines and incisors at the end of the active phase of orthodontic treatment [3,4]. The protocol of choice for most orthodontists is the bonded lingual retainer (BLR) (reported by 83% of the orthodontists asked) [5], since it is invisible, independent of patient cooperation and is aimed at lifelong retention.

Although BLR is widely used and has many benefits, it is prone to failures [6] and adverse negative effects, such as localized tooth movement, torque changes, and gingival recessions [6,7]. Long-term BLR follow up and maintenance by either the orthodontist or the treating general dental practitioner is crucial for long-term stability of the orthodontic treatment result and the health of the supporting periodontal tissues [8]. Therefore, all patients must be informed at the end of treatment about the need for frequent follow up visits as long as the BLR is bonded.

Gingival recession is defined as the displacement of the gingival margin apical to the cemento–enamel junction (CEJ) with exposure of the root surface to the oral environment [9]. Miller’s classification of gingival recessions is based on the presence or absence of keratinized tissue at the apical extent of the defect, proximal bone support, and the location of the tooth [10]. Miller’s class III is defined as marginal tissue recession, which extends to, or beyond the muco-gingival junction, either due to bone or soft tissue loss in the interdental area or due to malpositioning of the tooth. The main challenge of mucogingival therapy in Miller class III recessions is to obtain full root coverage.

It was suggested that different surgical procedures will provide a significant reduction in recession depth and root coverage of Miller class I and II tooth recession defects [10]; however, no coverage or only partial coverage can be expected in class III and IV defects [11].

The development of gingival recession during orthodontic treatment is associated with mechanics causing tooth movement out of its alveolar envelope (i.e., expansion, proclination or retroclination) [12,13]. However, a recent systematic review indicated that orthodontic treatment did not necessarily affect the development of gingival recession of mandibular incisors [14]. Yet, the etiology is different in the case of post-orthodontic movements. In these cases, inadvertent tooth movement might be caused by a non-passive retainer, distortion of the BLR during the bonding of a passive retainer, or by deformation of the BLR used for retention due to biting forces on hard foods or a consistent habit leading to activation of the retainer’s wire [8,15].

A cohort study on the development of labial gingival recessions in orthodontically treated patients, conducted by Renkema et al. [16], found that the prevalence of the labial gingival recessions increases with age and with the time passed since the retention period starting point. The study shows gingival recessions in 20% of the patients two years post-treatment and in 38% of the patients five years post-treatment.

The successful outcome of the root coverage procedure is to achieve stable gingival margin coronal to the cemento–enamel junction (CEJ), and soft tissue integration with adjacent tissue [17].

Moreover, treating Miller class III recessions should be aimed at achieving a satisfactory esthetic outcome for the patient and to resolve any hypersensitivity present with minimal patient morbidity [17,18,19].

Thus, the aim of this study was to explore the benefit of a combined periodontic-orthodontic approach to resolve Miller class III gingival recessions in post-orthodontic patients.

## 2. Materials and Methods

**Figure 1:**
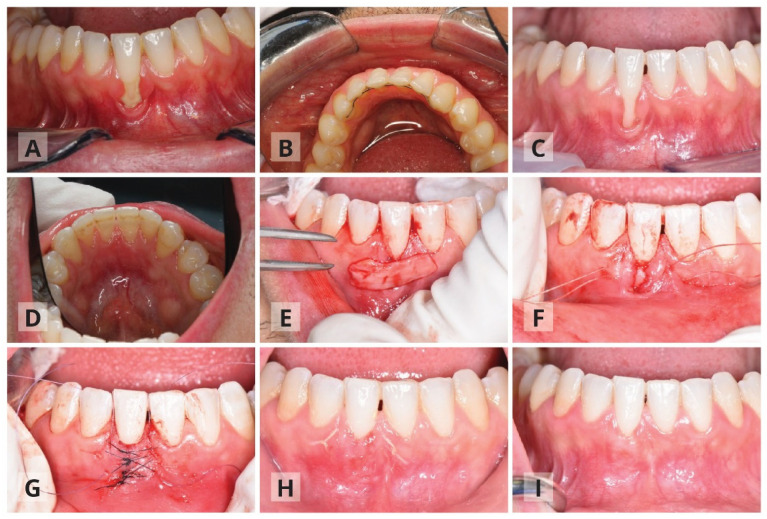
First protocol, which included bonded lingual retainer removal. Patient number thirteen—thirty-one-year-old male arrived with Miller class III recession in tooth #41. Ten years post-orthodontic treatment, with bonded lingual retainer present (**A**,**B**). Three months after removal of the bonded lingual retainer and prior to surgery (**C**,**D**). During the surgery, the tunneling procedure that includes insertion of connective tissue graft (**E**,**F**). Double papilla suturing and addition of coronal anchoring sutures (**G**). Gingival healing two weeks post-surgery (**H**) and three months post-surgery (**I**).

**Figure 2:**
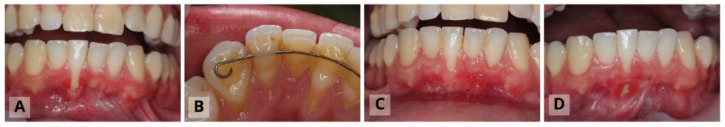
Second protocol—without bonded lingual retainer (BLR) removal. Patient number three— 27-year-old male arrived with Miller Class III recessions both on the buccal and the lingual surfaces of tooth #41. Eight years passed since orthodontic treatment completion and lingual fixed retainer was maintained. Lingual retainer was not removed prior to surgery (**A**,**B**). One month after surgery a gingival recession of 3 mm was present (**C**). Six months follow up after surgical procedure, note the gingival fenestration (**D**).

Fifteen cases from the periodontics department at Tel Aviv University were reviewed—all underwent orthodontic treatment and had a BLR on the day of the first examination. The study was approved by the ethical committee of Tel Aviv University (Authorization number 0001732-3). The cases were divided into two groups according to the protocol used: the first included removal of the BLR prior to surgery and a removable retainer was applied to the patient three months after surgery for night-use only (Figure 1). The second protocol did not include BLR removal, only a surgical approach (Figure 2). All patients underwent the same surgical procedure as detailed below (Figure 1). Prior to surgery, occlusal adjustments were made if needed to prevent traumatic occlusal forces. The gingival recession was measured using a North Carolina periodontal probe (UNC-15, Hu-Friedy, Chicago, IL, USA) before the surgery, and three to six months after. Vertical recession was measured at the mid-buccal aspect of the tooth from the cemento–enamel junction (CEJ) to the gingival margin. All cases were documented and photographed using a Canon d-70 camera.

### The Surgical Procedure: Modified Tunnel Double Papilla Procedure for Root Coverage

Composite flow (3M) was applied on the contact points mainly for the anchorage of the sutures and the coronally positioned flap, and to avoid unwanted tooth movements. The surgical procedure is detailed briefly; a partial thickness tunnel was prepared. The tunnel extended 3–4 mm apically beyond the muco-gingival junction and to the recession’s adjacent teeth mesially and distally, while undermining the facial aspect of the interdental papilla. The goal was to join the two edges of the recession with no tension and to allow easy coronal displacement of the flap. A connective tissue graft (CTG) was harvested according to Hürzeler and Weng [20] (Figure 1E,F). The connective tissue was inserted and stabilized with resorbable sutures and the two edges of the gingival recession were then approximated to be tension free, with simple interrupted sutures starting from the apical part of the recession. These sutures allow primary soft tissue closure over the connective tissue graft (Figure 1G). Buccal and lingual vertical sutures were added over the temporary splinted teeth for coronally flap positioning. The post-operative instructions included rinsing with 0.2% chlorhexidine (CHX) solution twice a day for two weeks; no anti-inflammatory medication was prescribed and patients were instructed to take analgesics of their preference when needed. Teeth brushing in the surgical area was discontinued during the first post-operative two weeks. Patients were instructed gradually to brush the area with an ultra-soft brush soaked in 0.2 CHX for another 2–6 weeks. Follow up was conducted once a week during the first two months, and professional tooth cleaning was also advised.

The surgical procedure was classified as successful if a reduction in the gingival recession was noted.

The statistical analysis was carried out using SPSS software for Windows (version 20; SPSS, Chicago, IL, USA) and significance level was set for *p* = 0.05. Non-parametric analysis (Mann–Whitney) was used to explore differences between the two studied groups.

## 3. Results

Out of the 15 cases that were analyzed, the first protocol that included removal of the BLR was applied to six patients and the second protocol was applied to the other nine patients (Table 1). There was no significant difference in the average age of the patients (*p* = 0.224) or the initial recession depth (*p* = 0.529) between the two groups.

A significant difference was found in the reduction in the recession depth between the two protocols used: the average improvement in recession depth for the first protocol was 4.0 ± 0.83 mm as compared to the second protocol which showed less improvement in recession depth with an average of 1.88 ± 1.29 mm (*p* = 0.008). Recession depth after surgery was significantly reduced in the first protocol (0.66 ± 0.60 mm) compared to the second protocol (2.33± 0.96 mm) (*p* = 0.008). The first protocol showed an improvement of 87.2% as compared to the second protocol which showed an improvement of 43.8% (*p* = 0.008).

## 4. Discussion

Tooth malposition post-orthodontic treatment leading to gingival recession is most often due to an unwanted force extracted by distortion and activation of the retainer’s wire. Therefore, the first stage of resolving and treating the gingival recessions is carried out to remove the BLR by either the orthodontist or the periodontist. This should be followed by immediate clinical documentation that is critical for follow-up changes.

Although only 15 cases were examined in the current study, the significant improvement in Miller class III recession treatment following the protocol that included BLR removal was striking. Most of the patients have completed the orthodontic treatment as young adults (between the ages of 15 and 18 years); however, no data regarding the exact starting time of the recession, nor the changes over time could be obtained accurately. All the patients reported fast progression of the recession, which caused them great concern and encouraged them to seek treatment urgently.

The issue of BLR deformation is mostly common in flexible spiral wires, which were used for the patients in our study. It was suggested that flexible spiral wires retainers bonded on all six mandibular anterior teeth might induce unexpected movement of anterior teeth to such an extent that retreatment is necessary [21]. These unexpected movements are not considered to be a relapse, as torque differences were not present either before or at the end of orthodontic treatment. Rather, it might be the result of wire deflection during bonding or mechanical deformation in the post-treatment period [21].

We assume that the removal of the BLR allows spontaneous correction of the tooth position due to the release of undesired forces. Such movements are the result of tension changes in the periodontal and gingival fibers, changes in occlusal forces, or soft tissue pressure (cheeks versus the tongue), which are limited by the bone envelope [2]. Apparently, these position changes improve the surgical results of the perioplastic surgery and its long-term stability. In cases treated using the second protocol in which the BLR was not removed prior to the surgical intervention, unwanted forces continued to affect tooth position and caused surgical procedure failure.

It should be noted that the removal of the BLR might contribute to the improvement in oral hygiene; however, its effect on the result of the surgery is minor as the hygiene of the buccal surface is not directly affected by lingual retainer location [22].

Orthodontic correction of roots positioned outside the alveolar envelope is important to reduce gingival recessions and to provide favorable conditions to the periodontal surgery [23]. Orthodontic retreatment will enhance the surgical result; however, it can be performed only when there is enough lingual bone support [24]. If orthodontic re-treatment is not feasible and/or refused by the patient, the removal of the BLR prior to the surgical procedure might improve the surgical outcome.

In this study, we used the Miller classification that became a common approach in identifying soft tissue recessions [10]. Recently, the use of Miller’s classification was reviewed and the difficulty in assignment of some recessions to a specific class was outlined [25,26]. Cairo et al. [27] suggested a new classification system of gingival recessions based on the interproximal clinical attachment level. Gingival recessions without loss of interproximal attachment were considered to be RT1 defects and with interproximal attachment loss were considered to be RT2 or RT3 defects. In the new classification by Cairo et al. [27], the malposition of the root is not considered a prognostic criterion, although it is a limiting factor for the amount of root coverage achieved at the buccal site after surgery. This may be associated with the blood supply provided by interproximal soft tissue to the buccal flap/graft during the healing process. Therefore, we find the consideration of the tooth position by Miller’s classification system more suitable for post-orthodontic lower incisors recessions.

Root coverage techniques applying a CTG appear to be the gold standard [28,29,30], showing a high degree of root coverage with excellent esthetic results [31] both in short- and long-term follow ups [32,33,34].

The envelope technique, where a CTG is placed underneath a split thickness flap around the recession [35], originally described for single-tooth cases, resulting in 84% mean root coverage with complete root coverage in 61% of the sites [36,37]. Several variations to this surgical approach were suggested later on: horizontal mattress sutures to mesially and distally anchor the CTG underneath the envelope [38], muco-periosteal-mucosal tunnel where a coronally positioned flap was anchored at the contact area with mattress sutures to cover the CTG [39] and the modified microsurgical tunnel technique [40]. It was suggested that in the case of post-orthodontic lower incisor recessions, the modified tunnel double papilla procedure should be used to obtain maximal root coverage [41].

## 5. Conclusions

Treating Miller class III recessions in post-orthodontic patients should include the elimination of unwanted exerted forces, which affects tooth position. The removal of the bonded lingual retainer prior to the surgical procedure showed significantly greater improvement in recession depth, compared to cases in which the retainer was kept. The removal of the lingual retainer in combination with the suggested surgical technique is recommended for improving root coverage in Miller class III recessions.

## Figures and Tables

**Table 1:** Description of patients who attended the study, including recessions depth (in mm) before and after the surgery. Recession type following Cairo et al. [17].

						Recession Depth		
Patient Number	Gender	Age	Tooth Number	Bonded Lingual Retainer Removal	Recession Type	First Examination (mm)	After Surgery	Improvement (%)
							(mm)	
1	Male	37	32	no	1	6	1	83.33
2	Male	30	41	no	2	3	0.5	83.33
3	Male	27	41	no	1	5	3	40
4	Female	30	31	no	1	4	2.5	37.5
5	Female	25	31	no	2	4	3	25
6	Female	42	31	no	2	4	3	25
7	Male	28	31	no	1	4	2	50
8	Female	25	31	no	1	4	3	25
9	Female	28	31	no	1	4	3	25
10	Female	32	41	yes	1	4	0	100
11	Female	56	41	yes	2	6	1.5	75
12	Female	30	41	yes	2	4	1	75
13	Male	31	41	yes	2	5	0.5	90
14	Male	43	31	yes	2	6	1	83.33
15	Male	24	32	yes	1	3	0	100
